# Damaging Effects of Bisphenol A on the Kidney and the Protection by Melatonin: Emerging Evidences from In Vivo and In Vitro Studies

**DOI:** 10.1155/2018/3082438

**Published:** 2018-02-18

**Authors:** Anongporn Kobroob, Wachirasek Peerapanyasut, Nipon Chattipakorn, Orawan Wongmekiat

**Affiliations:** ^1^Department of Physiology, Faculty of Medicine, Renal Physiology Unit, Chiang Mai University, Chiang Mai 50200, Thailand; ^2^Department of Physiology, Faculty of Medicine, Cardiac Electrophysiology Research and Training Center, Chiang Mai University, Chiang Mai 50200, Thailand

## Abstract

This study investigates the effects of bisphenol A (BPA) contamination on the kidney and the possible protection by melatonin in experimental rats and isolated mitochondrial models. Rats exposed to BPA (50, 100, and 150 mg/kg, i.p.) for 5 weeks demonstrated renal damages as evident by increased serum urea and creatinine and decreased creatinine clearance, together with the presence of proteinuria and glomerular injuries in a dose-dependent manner. These changes were associated with increased lipid peroxidation and decreased antioxidant glutathione and superoxide dismutase. Mitochondrial dysfunction was also evident as indicated by increased reactive oxygen species production, decreased membrane potential change, and mitochondrial swelling. Coadministration of melatonin resulted in the reversal of all the changes caused by BPA. Studies using isolated mitochondria showed that BPA incubation produced dose-dependent impairment in mitochondrial function. Preincubation with melatonin was able to sustain mitochondrial function and architecture and decreases oxidative stress upon exposure to BPA. The findings indicated that BPA is capable of acting directly on the kidney mitochondria, causing mitochondrial oxidative stress, dysfunction, and subsequently, leading to whole organ damage. Emerging evidence further suggests the protective benefits of melatonin against BPA nephrotoxicity, which may be mediated, in part, by its ability to diminish oxidative stress and maintain redox equilibrium within the mitochondria.

## 1. Introduction

Bisphenol A (BPA) is one of the oldest synthetic compounds known for its endocrine activity, although it is subsequently replaced by the stronger estrogenic activity of diethylstilbestrol. BPA is now used extensively as a starting material for epoxy resins lining food and beverage containers and as a monomer in polycarbonate and polysulfone-based plastics in a multitude of consumer products, including medical and dental devices [1]. Epidemiological studies have shown that over 90% of individuals tested had detectable levels of BPA, providing important evidence of ubiquitous and continual BPA exposure even in the general population [2]. Although published data regarding occupational exposure to BPA are limited and the potential for BPA-related health effects among the workers is unknown, recent study has shown the increased urinary total BPA concentrations in the group of manufacturing workers [3]. These data have raised concerns about the possible implication of BPA in the etiology of various human diseases [4]. Experiments using cultured cells and laboratory animals demonstrated that BPA is able to accumulate and affect several vital organ functions, including the testis [5], brain [6], heart [7], liver [8], and pancreas [9]. The findings also indicate an involvement of oxidative stress and mitochondrial dysfunction to the damaging effect of BPA [10–13].

Recently, emerging evidence from epidemiological studies has shown an association of high urine BPA levels and low-grade albuminuria in both adults and children [14, 15]. The relations between high serum BPA levels and increased oxidative stress and inflammatory markers in hemodialysis patients using BPA-containing polysulfone dialyzers have also been reported [16]. These findings called attention to the possibility that exposure to BPA during everyday life could have adverse effects on the kidney and might contribute to progressive cumulative renal injury over a lifetime. However, it remains to be determined whether these observations are indeed the certain consequences of BPA.

Given that nephrotoxicity is a significant public health concern as it can progress to chronic kidney disease in the long run, it is essential to explore whether BPA contamination could actually have an unfavorable effect on the kidney. The first study was, therefore, set up to address this issue as well as the potential contribution of oxidative stress and mitochondrial dysfunction in mediating BPA nephrotoxicity using an in vivo rat model that was repeatedly exposed to BPA. The second study was initiated based on the hypothesis that the use of antioxidant targeting on mitochondria may find therapeutic benefit in diminishing the detrimental cellular and organ outcomes of BPA. In this regard, melatonin (*N*-acetyl-5-methoxytryptamine), a compound derived from tryptophan, was selected as it has been shown to be a strong antioxidant having the capability of penetrating into the mitochondria and exerting protection against several disease conditions that are related to oxidative stress, including kidney diseases [17, 18]. The last experiment was conducted in vitro, using isolated kidney mitochondria, to verify that the renal consequences of BPA and melatonin were indeed produced by its direct action on mitochondria, causing mitochondrial dysfunction, oxidative stress, and, eventually, whole organ damage.

## 2. Materials and Methods

### 2.1. Drug and Chemicals

Thiopental was obtained from Ceva Animal Health Ltd. (Bangkok, Thailand). All other chemicals and reagents were of analytical grade and were purchased from Sigma-Aldrich Co. (St. Louis, MO, USA).

### 2.2. Animal Preparation

Male Wistar rats, weighing 180–200 g, were obtained from the National Laboratory Animal Center Mahidol University, Salaya, Thailand, and housed under standard temperature and humidity conditions with a 12 h light-dark cycle. Food and water were given ad libitum. The animals were allowed one week to acclimatize before starting the experiment. All procedures were conducted in conformity with the guidance for the use of animals by the National Research Council of Thailand and were approved by the Institutional Animal Care and Use Committee at the Faculty of Medicine, Chiang Mai University.

### 2.3. Experimental Designs

The present research work consisted of two main parts, the in vivo and in vitro studies. The in vivo studies included two independent experiments, which were carried out using rats repeatedly exposed to BPA for 5 weeks. Likewise, the in vitro studies comprised four different experiments, which were performed using mitochondria isolated from the kidneys of rats and subsequently subjected to direct BPA exposure.

#### 2.3.1. In Vivo Studies

The in vivo investigations were subdivided into two separate study protocols. The first protocol was undertaken as a preliminary study to examine the impacts of BPA contamination on the kidney and to identify the most appropriate dose of BPA to use in the subsequent protocol. The study also extended to evaluate whether oxidative stress and mitochondrial dysfunction underlie the renal effect of BPA. Rats were randomly divided into 4 groups (*n* = 4 each). Three of them were injected intraperitoneally with BPA at 50, 100, and 150 mg/kg/day, respectively, while the remaining group was given vehicle (corn oil) and used as control. The maximum dose of BPA was selected based on previous data indicating BPA-induced hepatotoxicity in rats [12], whereas the minimum dose chosen showed BPA-induced oxidative injury in rat testis [19]. The treatment lasted for 5 weeks. Food, water intake, and body weight were recorded on a daily basis. On the last treatment day, rats were placed in metabolic cages for 24 h urine collections. Blood samples and kidney tissues were taken thereafter through a midline laparotomy under thiopental anesthesia (80 mg/kg, i.p.) for determinations of renal function, oxidative stress, mitochondrial function, and histopathological studies.

In the second protocol, the renoprotective potential of melatonin against BPA toxicity was explored. Experiments were performed on three groups consisting of 8 rats each. Rats in group 1 (Veh) received vehicle only. Rats in group 2 (BPA) were injected intraperitoneally with BPA at 50 mg/kg/day (the appropriate dose obtained from the results of the first protocol). In addition to BPA, rats in group 3 (BPA + MEL) were cotreated with melatonin at the dose of 10 mg/kg/day for 5 weeks. Melatonin was injected intraperitoneally 30 min before BPA injection. The selected dose and regimen for melatonin treatment were based on previous reports showing its efficacy to prevent BPA toxicities on the cerebellum [20] and reproductive system [19, 21]. Renal function, oxidative stress, mitochondrial function, and histopathology were evaluated at the end of the experiment.

#### 2.3.2. In Vitro Studies

The in vitro studies were undertaken to test if BPA and melatonin act directly on mitochondria to produce its renal consequences. Four sets of experiments were carried out using the mitochondria that were isolated from the kidneys of rats. In the first experiment, the possible direct effects of BPA on kidney mitochondrial function were investigated. Isolated mitochondria were exposed to BPA at various concentrations, ranging from 0 to 1000 *μ*M for 15 min. The alterations in mitochondrial function were evaluated by detections of mitochondrial reactive oxygen species (ROS) production, mitochondrial membrane potential change (Δ*Ψm*), and mitochondrial swelling.

The second experiment aimed to determine the potential benefits of melatonin on isolated mitochondria that were exposed to BPA. The mitochondria were incubated with or without BPA and in the presence or absence of melatonin. Thereafter, changes in the mitochondrial function were evaluated. Various concentrations of melatonin (0.1–100 *μ*M) were tested, whereas only one proper concentration of BPA (125 *μ*M) based on the results of the first in vitro experiment was used. In case of melatonin pretreatment, the mitochondria were incubated with melatonin for 5 min before exposure to BPA for further 15 min.

The third experiment was carried out to examine whether the effects of BPA and melatonin were mediated through mitochondrial oxidative stress mechanisms. Four groups of isolated kidney mitochondria were studied. Group 1, the mitochondria were treated with vehicle and used as control. Group 2, the mitochondria were treated with melatonin at the dose which was shown in the second experiment to be the most effective one (0.5 *μ*M). Group 3, the mitochondria were treated with BPA (125 *μ*M). Group 4, the mitochondria were treated with both BPA (125 *μ*M) and melatonin (0.5 *μ*M). Mitochondrial oxidative stress was evaluated by determinations of lipid peroxidation and antioxidant glutathione levels.

The last experiment was carried out to examine the alterations in mitochondrial ultrastructure following BPA (125 *μ*M) and melatonin (0.5 *μ*M) treatments. Additional set of kidney mitochondria with the same experimental design as in the third in vitro study was reproduced, and transmission electron microscopic examination was used to demonstrate the change in mitochondrial ultrastructure.

### 2.4. Assessment of Renal Function

Renal function was assessed by determinations of blood urea nitrogen (BUN), serum creatinine, urine creatinine excretion, and urine protein excretion using AU 480 chemistry analyzer (Beckman Coulter Inc., Brea, CA, USA). Creatinine clearance, an index of glomerular filtration rate (GFR), was calculated from the ratio of creatinine in urine/serum and the volume of urine produced. A urine protein-to-creatinine ratio (UPCR) was also evaluated.

### 2.5. Assessment of Renal Oxidative Stress

Kidney tissues were homogenized in appropriate ice-cold buffers using a Potter Elvehjem homogenizer (Wheaton Science, Millville, NJ). The supernatant obtained after centrifugation (10,000*g*, 4°C, 15 min) was used for determinations of nitric oxide (NO), malondialdehyde (MDA), reduced glutathione (GSH), and superoxide dismutase (SOD) using commercial kits (BioAssay Systems, Hayward, CA, USA) according to the manufacturer's instructions.

### 2.6. Histopathological Studies

#### 2.6.1. Light Microscopic Studies

The kidney tissues were fixed in 10% neutral buffered formaldehyde, dehydrated through a graded alcohol series, cleared in xylene, and embedded in paraffin wax. Serial sections of 4 *μ*M were deparaffinized, hydrated, and stained with hematoxylin and eosin (H&E) for light microscopic examination.

#### 2.6.2. Electron Microscopic Studies

The kidney tissues (or mitochondrial pellets in case of in vitro studies) were fixed with 2.5% glutaraldehyde in 0.1 M phosphate buffer, pH 7.4 at 4°C overnight. After rinsing in phosphate buffer, the kidney tissues or mitochondrial pellets were postfixed in 2% phosphate-buffered osmium tetroxide for 2 h at room temperature, dehydrated in a graded series of ethanol, rinsed in propylene oxide, and embedded in Epon-Araldite. Ultrathin sections (60–80 nm thick) were cut with a diamond knife, placed on copper grids, stained with uranyl acetate and lead citrate [22], and examined using transmission electron microscope (JEM-2200 FS, JEOL, Tokyo, Japan).

### 2.7. Mitochondrial Studies

#### 2.7.1. Preparations of Mitochondrial Fractions and Mitochondrial Proteins

Mitochondrial fractions were prepared as previously described [23]. Briefly, the kidney tissues were suspended in cold lysis buffer (230 mM mannitol, 70 mM sucrose, 1 mM EDTA, and 10 mM Tris-HCl, pH 7.4) and homogenized, then the mitochondria were isolated by differential centrifugation. The final mitochondrial pellet was suspended in ice-cold respiration buffer containing 250 mM sucrose, 5 mM KH_2_PO_4_, 10 mM Tris-HCl, and 2 mg/mL BSA, pH 7.2. Protein content of the mitochondria was quantified by bicinchoninic acid (BCA) assay using bovine serum albumin as standard [24].

#### 2.7.2. Determination of Mitochondrial ROS Production

Mitochondrial ROS production was assayed using a fluorescent dye 2′,7′-dichlorofluorescein diacetate (DCFDA) as previously described [23]. The measurement is based on ROS-mediated conversion of a nonfluorescent 2′,7′-dichlorofluorescein diacetate to a highly fluorescent dichlorofluorescein (DCF). Briefly, the mitochondria were incubated with 2 *μ*M DCFDA at 25°C for 60 min. The DCF fluorescence was measured at 485 nm excitation and 530 nm emissions using a fluorescence microplate reader (Synergy™ HT, BIOTEK^®^ Instruments Inc., Vermont, USA). The ROS level was expressed as arbitrary units of fluorescence intensity of DCF.

#### 2.7.3. Determination of Mitochondrial Membrane Potential Change (Δ*Ψm*)

A lipophilic cationic fluorescence dye 5,5′,6,6′-tetrachloro-1,1′,3,3′-tetraethylbenzimi-dazocarbocyanine iodide (JC-1) was used to assess mitochondrial membrane potential changes. The dye accumulates and aggregates in intact membranes, while it exists as monomers rather than as aggregates with loss of membrane integrity [25]. The assay was performed according to the previous method described [23]. Briefly, the mitochondria were incubated with JC-1 for 30 min at 37°C, and fluorescence was measured at excitation/emission 535/590 nm for the aggregate form and 485/530 nm for the monomer form. The change in mitochondrial membrane potential was expressed in terms of red/green (aggregate/monomer) fluorescence intensity ratio, where a decrease in the ratio implies the dissipation of membrane potential and reflects mitochondrial depolarization.

#### 2.7.4. Determination of Mitochondrial Swelling

A light-scattering technique was used to detect mitochondrial swelling by measuring the change in absorbance of the mitochondria at 540 nm over 15 min [26]. Kinetic measurements were carried out every 1 min at 25°C using microplate reader (Synergy H4, BIOTEK Instruments Inc., Vermont, USA). A decrease in absorbance indicates mitochondrial swelling.

### 2.8. Statistical Analysis

Data are presented as means ± SEM. Comparisons of the differences were performed by a one-way analysis of variance (ANOVA) followed by Fisher's LSD for multiple comparisons or nonparametric Kruskal-Wallis as appropriate. Statistical significance was taken at *P* < 0.05. All analyses were performed using SPSS 16.0 (SPSS Inc., Chicago, IL).

## 3. Results

### 3.1. In Vivo Studies: Impacts of BPA Contamination on the Kidney in Wistar Rats

#### 3.1.1. Effects of Various Concentrations of BPA on Body Weight, Kidney Weight, and Food Intake

The effects of BPA exposure at various concentrations on body weight, kidney weight, and food intake were shown in Table 1. The initial body weight was very similar in all experimental groups. At the end of the experiment, all rats exposed to BPA had a significantly lower body weight gain but higher kidney weight/body weight ratio than those receiving vehicle. These changes, which seemed to be in a dose-dependent fashion, were observed though the amount of food intake in all groups examined was comparable.

#### 3.1.2. Effects of Various Concentrations of BPA on Renal Function and Histopathology

**Figure 1:**
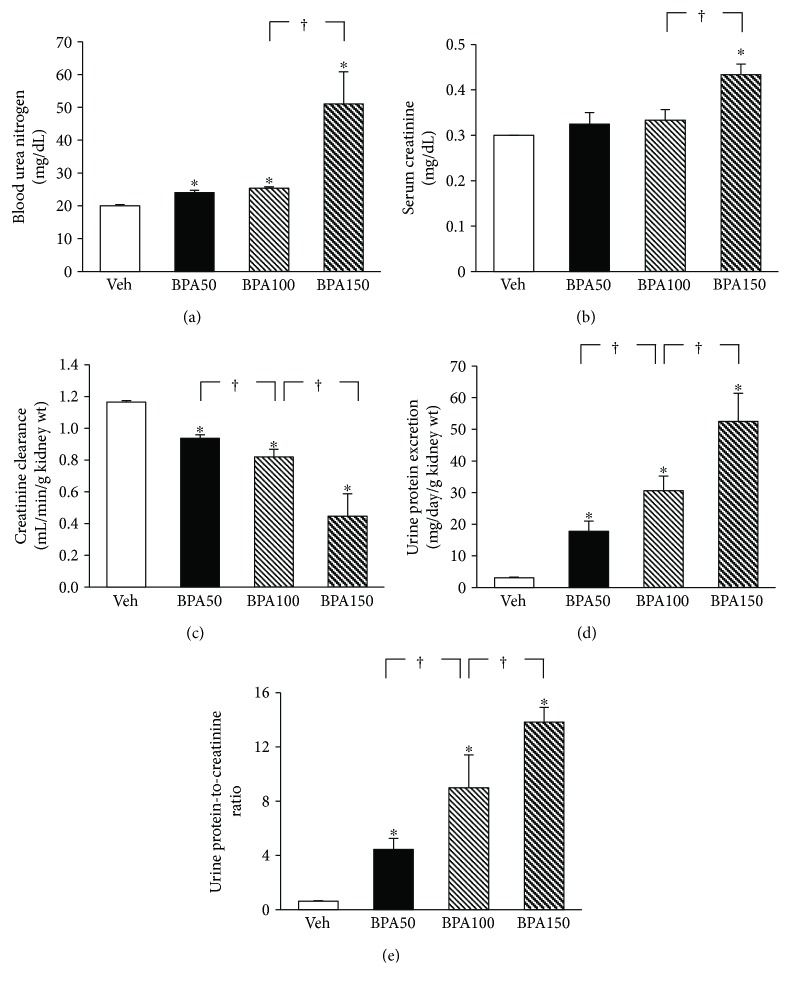
Effects of various concentrations of bisphenol A (BPA) on renal function. Values are means ± SEM (*n* = 4). Veh: vehicle-treated group; BPA (50, 100, and 150): BPA-treated group at 50, 100, and 150 mg/kg i.p., respectively, for 5 weeks. ^∗^*P* < 0.05 versus Veh, ^†^*P* < 0.05 between BPA-treated groups.

Exposure to BPA, irrespective of the doses, caused a significant increase in blood urea nitrogen (Figure 1(a)) compared to the vehicle control. The rise in serum creatinine (Figure 1(b)) was also observed in all BPA-treated groups, albeit the magnitude of increment reached statistically significant level only in the highest dose. A significant reduction in creatinine clearance (Figure 1(c)) but increases in urine protein excretion (Figure 1(d)) including urine protein-to-creatinine ratio (Figure 1(e)), in a dose-dependent manner, all coexisted after exposure to BPA.

**Figure 2:**
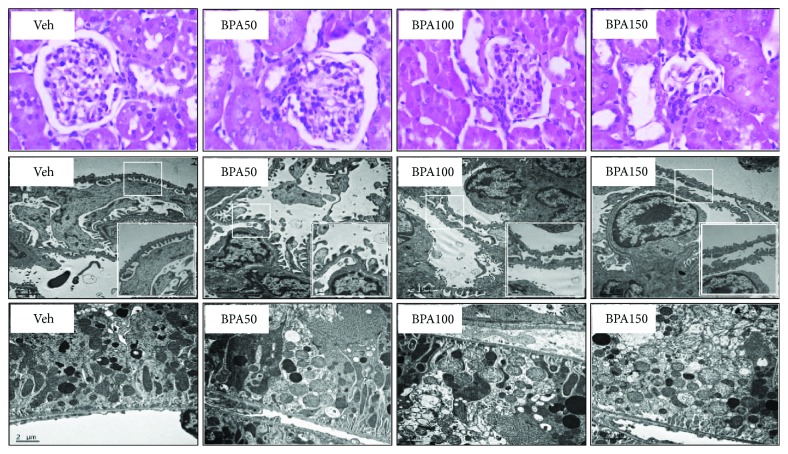
Photomicrographs of the kidney tissues following bisphenol A (BPA) exposure. Upper panel shows kidney sections stained with hematoxylin and eosin (H&E, 40x). Middle and lower panels show transmission electron micrographs of the glomerulus and renal tubules, respectively (original magnification: 3000x). The boxed areas are magnified in the right lower panel.

Light microscopic examinations of the kidney tissues in all experimental groups were depicted in Figure 2 (upper panel). Photomicrograph of the kidney from vehicle-treated group showed essentially normal architecture. By contrast, all rats contaminated with BPA exhibited a variety of glomerular injuries with increasing BPA concentration, ranging from hilar hyperplasia and mesangial proliferation in the low dose to glomerular sclerosis and atrophy in the highest dose, respectively.

BPA-induced glomerular injury was further supported by transmission electron microscopic examination (Figure 2, middle panel). Normal shape of podocytes with an intact glomerular basement membrane was observed in the vehicle group, while those treated with BPA, regardless of the dosage, displayed irregular podocytes, that is, cytoplasmic enlargement, foot process effacement, and glomerular basement membrane disruption.

The ultrastructure of renal proximal tubules was also demonstrated in Figure 2 (lower panel). Normal mitochondrial architecture with regular cristae was observed in rats given vehicle. In contrast, rats treated with BPA demonstrated mitochondrial swelling and loss of cristae. The severities were increased with increasing dose of BPA.

#### 3.1.3. Effects of Various Concentrations of BPA on Renal Oxidative Stress and Mitochondrial Function

**Figure 3:**
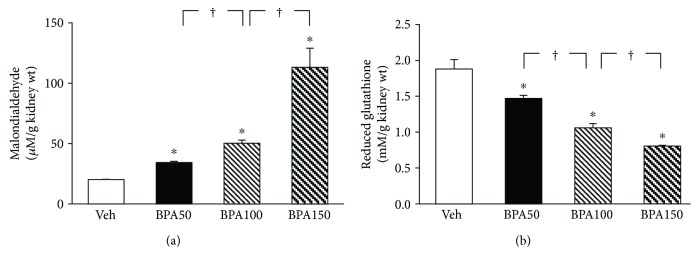
Effects of various concentrations of bisphenol A (BPA) on renal oxidative stress. Values are means ± SEM (*n* = 4). Veh: vehicle-treated group; BPA (50, 100, and 150): BPA-treated group at 50, 100, and 150 mg/kg i.p., respectively, for 5 weeks. ^∗^*P* < 0.05 versus Veh, ^†^*P* < 0.05 between BPA-treated groups.

A rise in the levels of MDA (Figure 3(a)) together with a dramatic fall in the levels of reduced glutathione (Figure 3(b)) in the kidney tissues was observed in all BPA-exposed rats compared to that in the vehicle control group (all *P* < 0.05). The magnitude of these changes was augmented with increasing dose of BPA.

**Figure 4:**
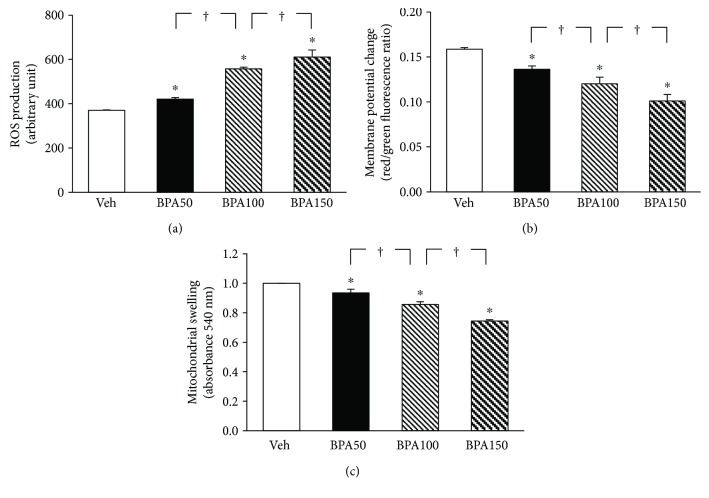
Effects of various concentrations of bisphenol A (BPA) on renal mitochondrial function. Values are means ± SEM (*n* = 4). Veh: vehicle-treated group; BPA (50, 100, and 150): BPA-treated group at 50, 100, and 150 mg/kg i.p., respectively, for 5 weeks. ^∗^*P* < 0.05 versus Veh, ^†^*P* < 0.05 between BPA-treated groups.

The mitochondria of rats exposed to BPA for 5 weeks exhibited a stepwise increase (*P* < 0.05) in the ROS production as BPA concentration increased (Figure 4(a)). A significant and dose-dependent reduction in membrane potential changes was also evident in the mitochondria of all BPA-treated groups as indicated by a decrease in JC-1 red/green fluorescence intensity ratio (Figure 4(b)). BPA contamination also caused the swelling of mitochondria as observed by a decrease in mitochondrial absorbance at 540 nm (Figure 4(c)), and the degree of swelling was gradually amplified when the BPA concentration was increased (all *P* < 0.05).

Overall, the results of this preliminary study revealed that BPA contamination at all concentrations (50, 100, and 150 mg/kg) examined caused renal injury. As BPA at 50 mg/kg was shown to be the lowest dose that consistently caused renal impairment with moderate degree of injury, thus, BPA at this concentration was selected for further investigation.

### 3.2. In Vivo Studies: The Renoprotective Potential of Melatonin in BPA-Exposed Rats

#### 3.2.1. Effects of Melatonin on Body Weight, Kidney Weight, Food Intake, Renal Function, and Histopathology in BPA-Exposed Rats

Compatible with the results obtained from the preliminary study, BPA-contaminated rats (50 mg/kg) gained weight less than those in the controls, while the ratio of kidney weight to body weight was higher (*P* < 0.05). These conditions were significantly improved in rats contaminated with BPA and concurrently treated with melatonin (Table 2).

**Figure 5:**
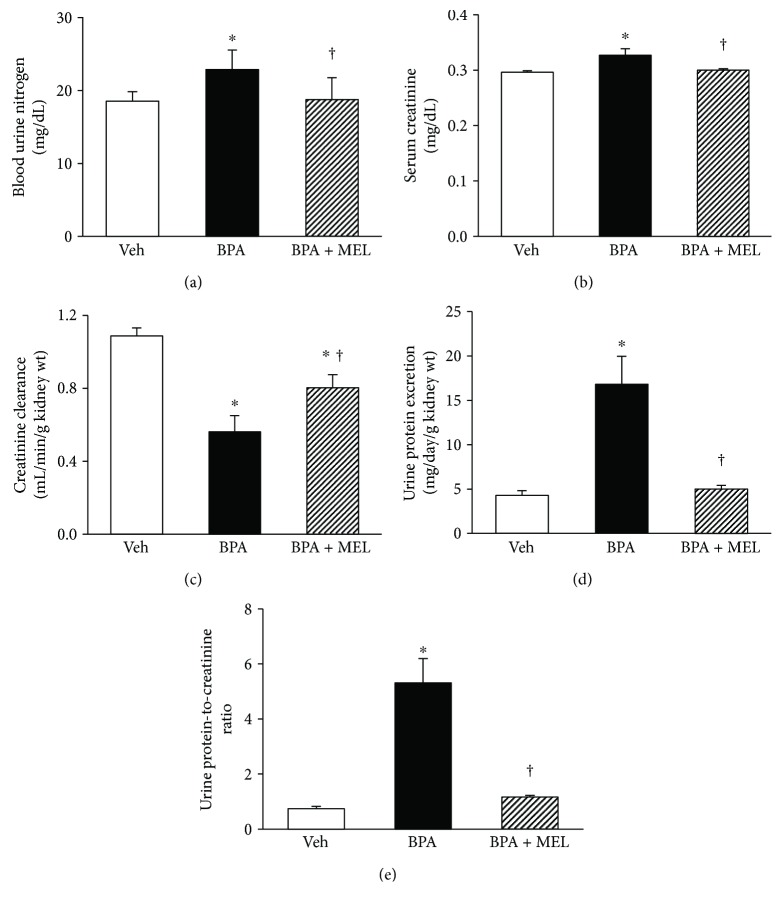
Effects of melatonin treatment on renal function in BPA-exposed rats. Values are means ± SEM (*n* = 8). Veh: vehicle-treated group; BPA: bisphenol A-treated group at 50 mg/kg, i.p.; BPA + MEL: BPA- (50 mg/kg, i.p.) plus melatonin- (10 mg/kg, i.p.) treated group for 5 weeks. ^∗^*P* < 0.05 versus Veh, ^†^*P* < 0.05 versus BPA.

**Figure 6:**
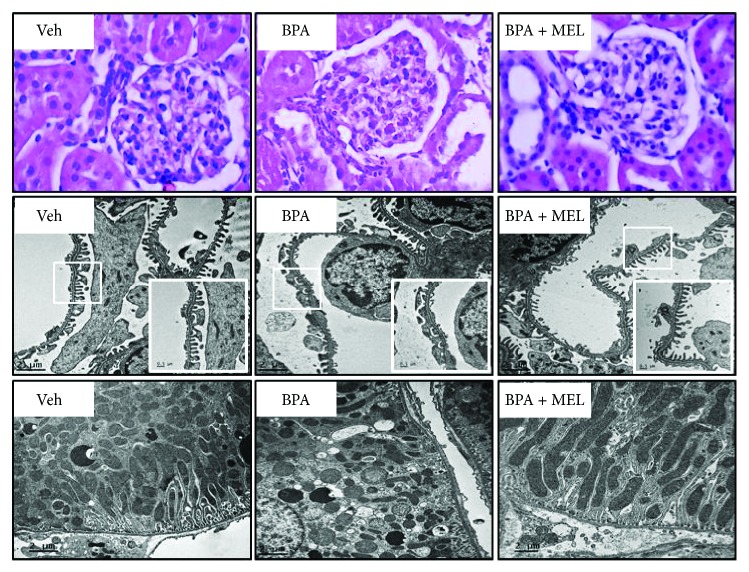
Photomicrographs of the kidney tissues treated with melatonin prior to bisphenol A (BPA) exposure. Upper panel shows kidney sections stained with hematoxylin and eosin (H&E, 40x). Middle and lower panels show transmission electron micrographs of the glomerulus and renal tubules, respectively (Original magnification: 3000x). The boxed areas are magnified in the right lower panel.

The rise in blood urea nitrogen (Figure 5(a)) and serum creatinine (Figure 5(b)) was significantly blunted when given melatonin alongside BPA. Melatonin also significantly attenuated BPA-induced fall in creatinine clearance (Figure 5(c)) and the presence of proteinuria (Figures 5(d) and 5(e)). Consistent with renal function, BPA-induced renal structural changes observed by light and electron microscopes were markedly improved following melatonin treatment (Figure 6). Focusing on renal tubules, electron micrographs were also able to demonstrate the well-preserved mitochondrial structure in the group treated with melatonin compared to that of BPA alone-treated group (Figure 6, lower panel).

#### 3.2.2. Effects of Melatonin on Renal Oxidative Stress and Mitochondrial Function in BPA-Exposed Rats

**Figure 7:**
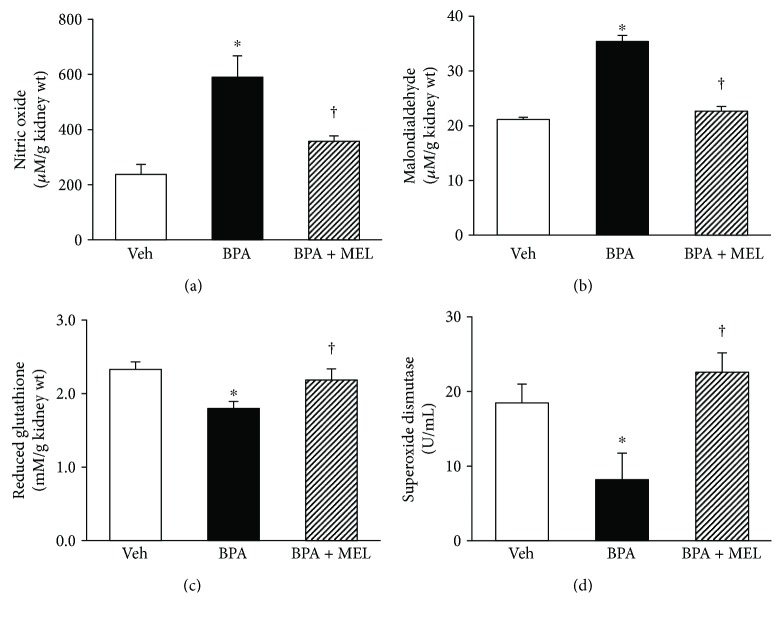
Effects of melatonin treatment on renal oxidative stress in BPA-exposed rats. Values are means ± SEM (*n* = 6). Veh: vehicle-treated group; BPA: bisphenol A-treated group at 50 mg/kg, i.p.; BPA + MEL: BPA- (50 mg/kg, i.p.) plus melatonin- (10 mg/kg, i.p.) treated group for 5 weeks. ^∗^*P* < 0.05 versus Veh, ^†^*P* < 0.05 versus BPA.

In view of renal oxidative stress (Figure 7), a striking increase in nitric oxide and malondialdehyde, together with a remarkable decrease in both nonenzymatic antioxidant glutathione and antioxidant enzyme superoxide dismutase, was observed after BPA (50 mg/kg) exposure (all *P* < 0.05). Supplementation of melatonin restored all the changes caused by BPA to the levels that were comparable to the controls (all *P* < 0.05).

**Figure 8:**
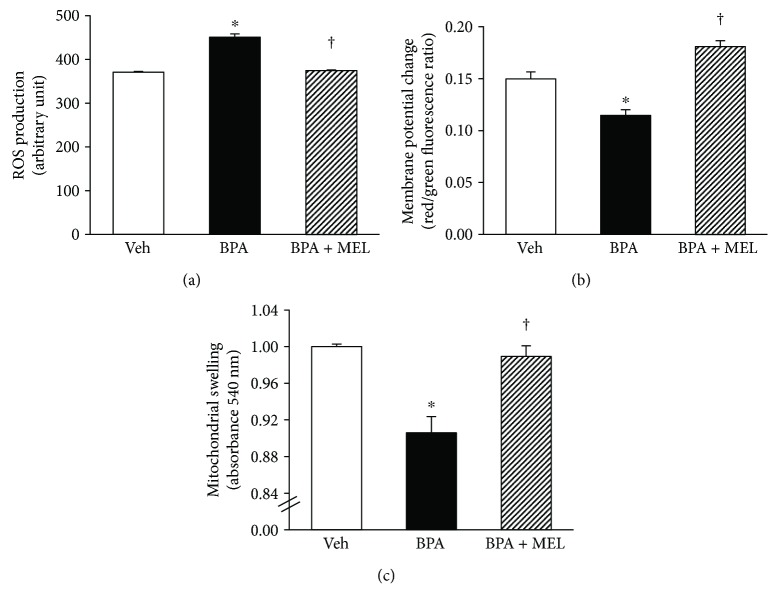
Effects of melatonin treatment on renal mitochondrial function in BPA-exposed rats. Values are means ± SEM (*n* = 6). Veh: vehicle-treated group; BPA: bisphenol A-treated group at 50 mg/kg, i.p.; BPA + MEL: BPA- (50 mg/kg, i.p.) plus melatonin- (10 mg/kg, i.p.) treated group for 5 weeks. ^∗^*P* < 0.05 versus Veh, ^†^*P* < 0.05 versus BPA.

Mitochondrial functional changes in response to BPA (50 mg/kg) and melatonin (10 mg/kg) treatments were demonstrated in Figures 8. Significant increases in ROS production but decreases in membrane potential changes were evident in the kidney mitochondria of rats exposed to BPA for 5 weeks. The swelling of the kidney mitochondria was also apparent in rats after 5 weeks of BPA exposure. These alterations in mitochondrial function caused by BPA were restored with melatonin treatment (all *P* < 0.05). These results were in agreement with the findings from electron microscopic studies as mentioned earlier (Figure 6, lower panel).

### 3.3. In Vitro Studies: Direct Impacts of BPA and Melatonin on Isolated Kidney Mitochondria

#### 3.3.1. Effects of Direct BPA Exposure on Isolated Kidney Mitochondrial Function

**Figure 9:**
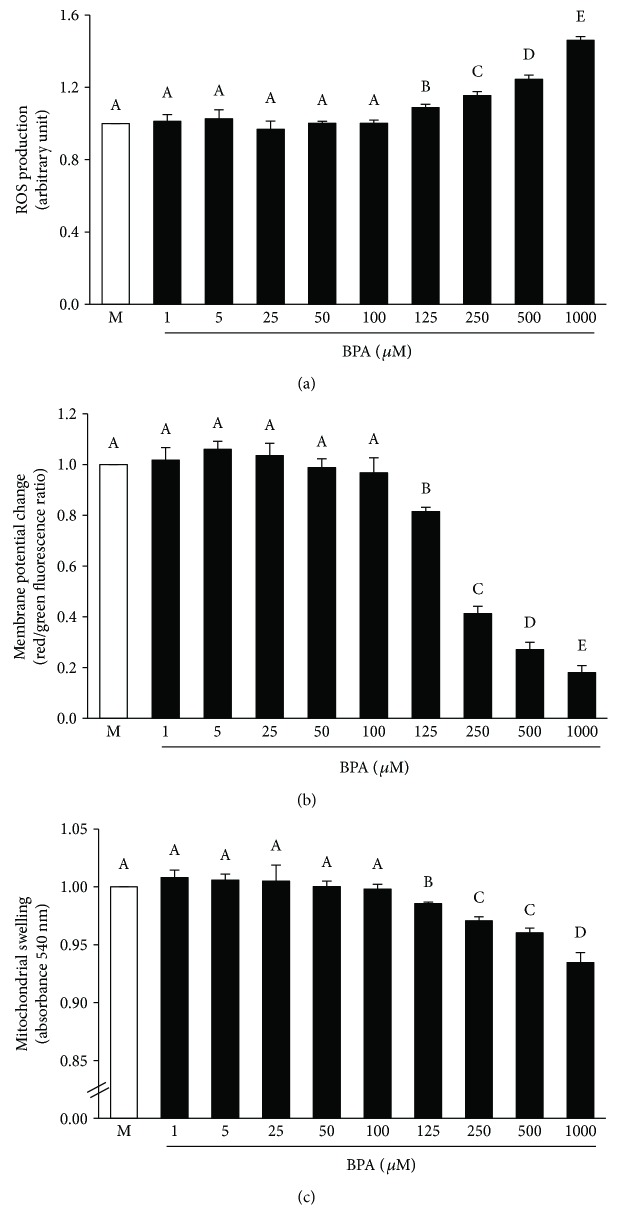
Effects of various concentrations of bisphenol A on isolated kidney mitochondrial function. Values are means ± SEM from the 5 experiments. M: vehicle-treated mitochondria; BPA (1, 5, 25, 50, 100, 125, 250, 500, and 1000): bisphenol A-treated mitochondria at 1, 5, 25, 50, 100, 125, 250, 500, and 1000 *μ*M, respectively. ^a–e^Different letters denote significant difference at *P* < 0.05.

Exposure of BPA at various concentrations (1–1000 *μ*M) directly to the isolated mitochondria resulted in a significant increase in mitochondrial ROS production (Figure 9(a)) in association with a dramatic fall in mitochondrial membrane potential change (Figure 9(b)). The mitochondria exposed to BPA also demonstrated mitochondrial swelling as shown by an absorbance reduction at 540 nm (Figure 9(c)). The impacts of BPA on the isolated kidney mitochondria appeared to be in a dose-dependent manner, which the initial effect was significantly detected at BPA 125 *μ*M. Therefore, BPA at this concentration was selected for further investigation in all the remaining protocols.

#### 3.3.2. Effects of Melatonin on Isolated Kidney Mitochondrial Function Following BPA Exposure

**Figure 10:**
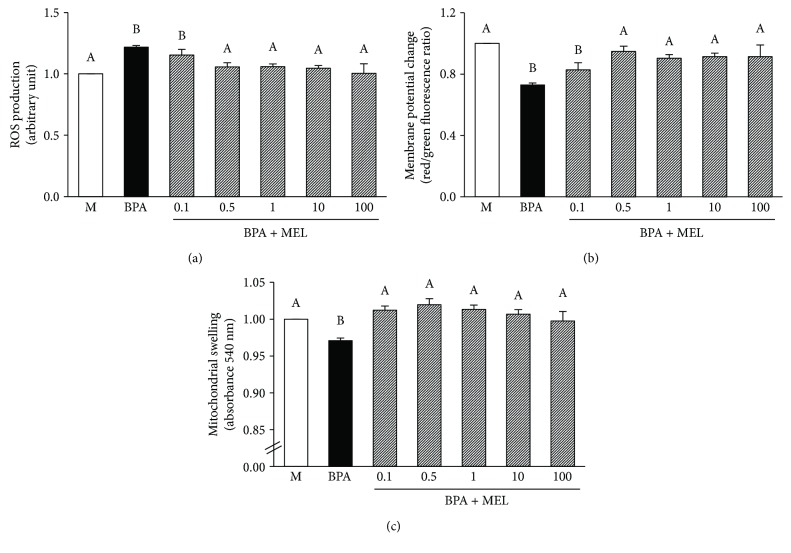
Effects of various concentrations of melatonin on isolated kidney mitochondrial function following BPA exposure. Values are means ± SEM from the 5 experiments. M: vehicle-treated mitochondria; BPA: bisphenol A- (125 *μ*M) treated mitochondria; BPA + MEL: melatonin-treated mitochondria at 0.1, 0.5, 1, 10, and 100 *μ*M, respectively, prior to BPA exposure. ^a–b^Different letters denote significant difference at *P* < 0.05.

The mitochondria isolated from the kidneys and pretreated with melatonin (0.1–100 *μ*M) were able to sustain their functions upon exposure to 125 *μ*M BPA (Figure 10). The protection by melatonin was significantly observed at all concentrations examined. However, it appears that the lowest dose (0.1 *μ*M) of melatonin showed the least benefit, particularly on its effect to reduce BPA-induced mitochondrial ROS production, when compared to all other doses (Figure 10(a)). Moreover, there were no significant differences in the benefits of melatonin with the increasing dose from 0.5 to 100 *μ*M. Thus, melatonin at 0.5 *μ*M was used in further study of mitochondrial oxidative stress and morphological changes.

#### 3.3.3. Effects of Melatonin on Isolated Mitochondrial Oxidative Stress Following BPA Exposure

**Figure 11:**
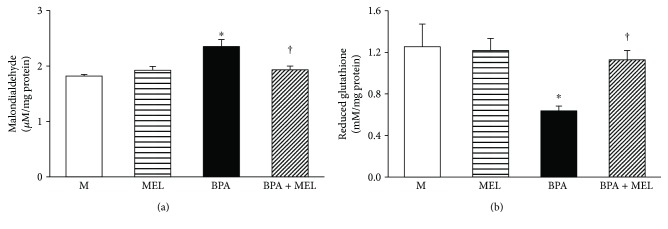
Effects of melatonin on isolated kidney mitochondrial oxidative stress following BPA exposure. Values are means ± SEM from the 5 experiments. M: vehicle-treated mitochondria; MEL: melatonin-treated mitochondria at 0.5 *μ*M; BPA: bisphenol A- (125 *μ*M) treated mitochondria; BPA + MEL: melatonin- (0.5 *μ*M) treated mitochondria prior to BPA exposure. ^∗^*P* < 0.05 versus M, ^†^*P* < 0.05 versus BPA.

The isolated mitochondria exposed to BPA (125 *μ*M) showed a significant increase in MDA levels (Figure 11(a)) and a marked reduction in nonenzymatic antioxidant glutathione (Figure 11(b)). Pretreatment with melatonin (0.5 *μ*M) completely prevented mitochondria from all the changes caused by BPA (all *P* < 0.05). None of these parameters were significantly altered in the group of mitochondria treated with melatonin alone compared to those in the control.

#### 3.3.4. Effects of Melatonin on Isolated Mitochondrial Ultrastructure Following BPA Exposure

**Figure 12:**
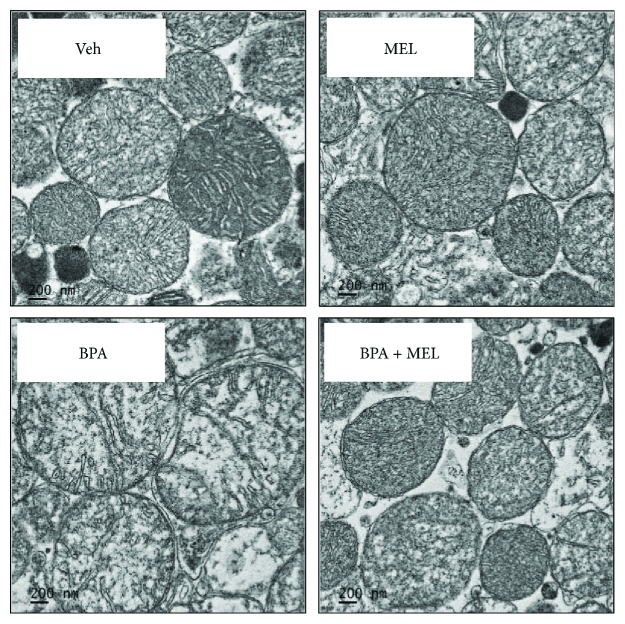
Transmission electron micrographs of isolated kidney mitochondria. Veh: vehicle-treated mitochondria; MEL: melatonin-treated mitochondria at 0.5 *μ*M; BPA: bisphenol A- (125 *μ*M) treated mitochondria; BPA + MEL: melatonin- (0.5 *μ*M) treated mitochondria prior to BPA exposure. Original magnification 15,000x.

Electron microscopic examination showed a normal structure of the control mitochondria with tight cristae and matrix (Figure 12(a)). Melatonin itself did not alter the mitochondrial configuration (Figure 12(b)). Following BPA exposure (125 *μ*M), the mitochondria appeared swollen, had less dense matrix, and had loss of cristae (Figure 12(c)). All structural alterations caused by BPA were attenuated when melatonin (0.5 *μ*M) was applied prior to BPA exposure (Figure 12(d)).

## 4. Discussion

The major issue challenged in this study is whether contamination with BPA could really have an adverse effect on the kidney. The current investigation also broadened to evaluate the efficacy of melatonin in the protection against any renal consequences that could ensue from BPA. The findings arise from this study confirm the impact of BPA in the kidney and also provide new insight regarding the potential therapeutic benefit of melatonin in diminishing the detrimental cellular and organ outcomes of BPA.

All rats in the BPA-treated groups showed less body weight gain than those in the vehicle-treated controls, and the severity was increased once the dose of BPA increased. This unusual body weight gain in the BPA-exposed rats may be affected by the reduction of food intake and/or the increase in energy expenditure. Although there was a tendency towards decreasing food intake upon increasing BPA exposure, the amount of food intake recorded from the BPA-treated groups was not significantly different from that obtained from the vehicle controls. The results suggested a catabolic effect of BPA rather than a disturbance of feeding mechanism. However, the renal hypertrophy did occur in BPA-contaminated rats despite the lower rate of growth development. This was evidenced by a dose-dependent increase in kidney weight when normalized to the corresponding body weight. These effects of BPA were mitigated by melatonin treatment.

The present study demonstrated that repeated exposure to BPA for 5 weeks resulted in azotemia, as indicated by increases in blood urea nitrogen and serum creatinine levels. A dose-dependent reduction in urine creatinine excretion including creatinine clearance was also evident in all rats contaminated with BPA. These results reflect that BPA has a negative impact on the kidney and leads to a deterioration of renal function. The impaired ability to excrete waste products can be caused by a defect in glomerular filtration and/or tubular function. Interestingly, a remarkably increased urine protein excretion and urine protein-to-creatinine ratio (UPCR), in a dose-dependent manner, were also coexisted after exposure to BPA. Since glomerular epithelial cells (podocytes) are commonly affected in proteinuric conditions, it is suggested that the renal functional impairment observed in this study may lie in the damaging action of BPA on the kidney glomeruli. This suggestion was supported by light microscopic studies showing a range of glomerular abnormalities upon BPA exposure and further confirmed by the observations of irregular podocyte foot processes (effacement) from the electron microscopy. These results correlated well with the previous study demonstrating BPA-induced podocytopathy and proteinuria in mice as well as a diminished expression of the slit diaphragm proteins nephrin and podocin in cultured podocytes [27]. The finding from electron microscopy also showed renal proximal tubular damage in BPA-treated groups as observed by the alteration in mitochondrial ultrastructure in a dose-dependent manner. Taken together, it is concluded that the impairment of renal tubular function following BPA exposure is the result of both BP-induced glomerular and tubular dysfunctions.

Oxidative stress is an imbalance between the production of reactive oxygen species and antioxidant defenses, causing oxidative damage [28]. The present study found that BPA exposure not only increased the oxidant molecule nitric oxide (NO) but also decreased the antioxidants glutathione (GSH) and superoxide dismutase enzyme (SOD) in the kidney tissues. This imbalance led to renal oxidative stress and, subsequently, renal oxidative damage as shown by an increase lipid peroxidation index, the malondialdehyde (MDA). These results indicate that BPA-provoked renal injury in this study was mediated through oxidative stress mechanism. Consistent with the current study, BPA has previously been shown both in vitro and in vivo to induce oxidative injury in a variety of cells and organs, that is, the liver [10], testes [5], and pancreas [9]. The relations between high serum BPA levels and increased oxidative stress markers have also been reported in hemodialysis patients using BPA-containing polysulfone dialyzers [16].

The mitochondria have been recognized as an important intracellular source of reactive oxygen (ROS) and nitrogen species (RNS) generation and defective mitochondria have been related to different pathological conditions [27]. The present investigation showed that rats repeatedly contaminated with BPA (at 50, 100, and 150 mg/kg) for 5 weeks exhibited renal mitochondrial dysfunction as supported by increased mitochondrial ROS production, decreased mitochondrial membrane potential, and induction of mitochondrial swelling. The magnitude of these changes was augmented with the increasing dose of BPA. Importantly, the present study was also able to demonstrate a dose-dependent impairment in mitochondrial function when BPA was applied directly onto the isolated mitochondria. This finding suggested that BPA can act directly on the kidney mitochondria to worsen its function. The present experiment in an in vitro model further demonstrated the incidence of BPA-induced mitochondrial oxidative stress, as indicated by increasing the levels of mitochondrial MDA while decreasing antioxidant glutathione. Collectively, current evidence suggests that the renal consequences of BPA, which occurred at a very high BPA exposure, are most probably produced via its direct action on the mitochondria, causing mitochondrial dysfunction, oxidative stress, and, eventually, whole organ damage. This suggestion was further supported by the previous study which showed that giving BPA to rats at 150, 250, and 500 mg/kg for 14 days resulted in hepatotoxicity [12]. The study also demonstrated that this dose-dependent induction of hepatotoxicity was produced by enhancing oxidative stress and impairing enzyme activities of the mitochondrial electron transport chain, and, thus, mitochondria have been indicated as an important target of BPA [12].

The present investigation revealed that coadministration of melatonin to the BPA-contaminated rats was able to prevent BPA-induced nephrotoxicity, as supported by the improvements in azotemia, proteinuria, mitochondrial functional impairment, and renal structural changes. The protection by melatonin was observed in association with the restorations of all oxidative parameters (NO, GSH, SOD, and MDA) in the kidney tissues, suggesting that melatonin exerted its renoprotection via antioxidant mechanism. This is compatible with several publications showing the free-radical scavenging and antioxidant properties of melatonin [17, 18, 29]. It has been demonstrated that melatonin reduces the activity of both constitutive and inducible nitric oxide synthase, the enzymes involved in the production of the potentially toxic nitric oxide [17, 30]. Melatonin also stimulates the activity and mRNA expression of glutathione peroxidase and increases the availability of glutathione, in addition to the increases in other antioxidant enzyme activities [29].

Importantly, the findings obtained herein further suggested that the mitochondrial protection by melatonin was mediated directly at the mitochondrial level. This suggestion arises from the present in vitro evidence showed that administration of melatonin directly onto the mitochondria prior to BPA exposure was able to protect BPA-induced mitochondrial dysfunction by the reduction of mitochondrial MDA and elevation of mitochondrial GSH contents. Consistent with these findings, the mitochondria have been identified as a target for melatonin actions, and melatonin has been found to be protective against mitochondrial oxidative damage under various pathological conditions [17, 31]. There are also reports showing that melatonin interacts with lipid bilayers, reducing lipid peroxidation and stabilizing mitochondrial inner membrane, thereby improving electron transport chain (ETC) activity [32]. A direct role for melatonin to maintain mitochondrial homeostasis, at least in part, to an effect on the expression of mtDNA has increasingly yielded considerable interest. Data have shown that melatonin could interact with complex I and IV of the ETC to increase complex activities and the subsequent promotion of electron flux through the ETC, resulting in increased ATP production [31–33]. However, whether these mechanisms also contribute to the protective effects of melatonin on renal mitochondria following BPA contamination observed in this study remained to be determined.

## 5. Conclusions

In conclusion, the present study provides convincing evidence to indicate that BPA has a detrimental impact on the kidney. Emerging evidences also underscore the mitochondrial oxidative stress as a key mediator in BPA-induced nephrotoxicity. The study outcomes further demonstrate the renoprotective benefit of melatonin against BPA toxicity which is mediated, at least in part, by diminishing oxidative stress and maintaining redox equilibrium within the mitochondria. However, further study is needed to explore the in-depth mechanism underlying the therapeutic efficacy of melatonin in combatting renal disorders elicited by this important industrial chemical. Besides, the current study assesses the adverse effects of BPA at high doses which are mostly being obtained by occupational exposure. Further study using low dose for longer time in order to reproduce a realistic scenario of exposure in general populations is also required.

## Figures and Tables

**Table 1:** Effects of various concentrations of bisphenol A on body weight, kidney weight, and food intake.

Experimental groups	Initial BW (g)	Food intake (g/day)	BW gain (%)	KW/BW (^∗^100)
Veh	237.00 ± 4.79	21.21 ± 0.83	56.87 ± 6.10	0.53 ± 0.02
BPA50	232.50 ± 9.46	20.29 ± 0.48	49.56 ± 5.32	0.60 ± 0.01^∗^
BPA100	242.50 ± 2.50	19.04 ± 1.28	30.96 ± 2.78^∗†^	0.68 ± 0.03^∗^
BPA150	235.00 ± 11.90	18.04 ± 2.85	12.95 ± 7.14^∗†#^	0.80 ± 0.06^∗^^†^

Data are presented as the mean ± SEM (*n* = 4). Veh: vehicle-treated group; BPA (50, 100, and 150): bisphenol A-treated group at 50, 100, and 150 mg/kg i.p., respectively, for 5 weeks; BW: body weight; KW: kidney weight. ^∗^*P* < 0.05 versus Veh, ^†^*P* < 0.05 versus BPA50, ^#^*P* < 0.05 versus BPA100.

**Table 2:** Effects of melatonin on body weight, kidney weight, and food intake in BPA-exposed rats.

Experimental groups	Initial BW (g)	Food intake (g/day)	BW gain (%)	KW/BW (^∗^100)
Veh	231.88 ± 1.32	22.14 ± 0.59	57.65 ± 2.83	0.61 ± 0.01
BPA	233.13 ± 2.66	21.29 ± 0.59	46.04 ± 2.97^∗^	0.78 ± 0.04^∗^
BPA + MEL	231.88 ± 2.98	25.50 ± 0.44^†^	54.16 ± 2.18^∗†^	0.72 ± 0.01^∗†^

Data are presented as the mean ± SEM (*n* = 8). Veh: vehicle-treated group; BPA: bisphenol A-treated group at 50 mg/kg i.p.; BPA + MEL: - (50 mg/kg, i.p.) plus melatonin- (10 mg/kg, i.p.) treated group for 5 weeks; BW: body weight; KW: kidney weight. ^∗^*P* < 0.05 versus Veh, ^†^*P* < 0.05 versus BPA.
